# The legacy of Malcolm Beverley Segal (1937–2019) on the science and fields concerned with choroid plexus and cerebrospinal fluid physiology

**DOI:** 10.1186/s12987-019-0161-6

**Published:** 2019-12-19

**Authors:** Adam Chodobski, Jean-François Ghersi-Egea, Jane Preston-Kennedy, Zoran Redzic, Nathalie Strazielle, Joanna Szmydynger-Chodobska, Robert G. Thorne

**Affiliations:** 1 Neurotrauma and Brain Barriers Research Laboratory, Department of Emergency Medicine, Alpert Medical School of Brown University, Providence, RI USA; 2 FLUID Team and BIP Facility, Lyon Neuroscience Research Center, INSERM, Lyon, France; 3 Institute of Pharmaceutical Science, King’s College London, London, UK; 4 Department of Physiology, Faculty of Medicine, Kuwait University, Jabriya, Kuwait; 5 Brain-i, Lyon, France; 6 Denali Therapeutics, South San Francisco, CA USA; 7 Department of Pharmaceutics, University of Minnesota, Minneapolis, USA

## Abstract

This article highlights the scientific achievements, professional career, and personal interactions of Malcolm B. Segal who passed away in July this year. Born in 1937 in Goodmayes, Essex, UK, Segal rose to the Chairman position in the Division of Physiology at United Medical and Dental School of Guy’s and St. Thomas’ Hospitals, retiring in 2006 after his long professional career in biomedical science. Being trained in Hugh Davson’s laboratory, Segal became one of the pioneers in research on cerebrospinal fluid physiology and the choroid plexus. During the course of his career, Segal himself trained a number of young scientists and collaborated with many colleagues around the world, making long-lasting friendships along the way. In addition to his professional accomplishments as a researcher and educator, Segal was an avid sailor and wine connoisseur, and enjoyed teaching classes on navigation and wine tasting.

Dr. Malcolm Beverly Segal, our colleague and dear friend, passed away after a long illness on the 29th of July 2019. Malcolm was the Chairman of the Division of Physiology at United Medical and Dental School of Guy’s and St. Thomas’ Hospitals and, following the merger of UMDS and King’s College London in 2000, he remained in the Department of Physiology until his retirement in 2006.

Malcolm was a pioneering figure in the field of cerebrospinal fluid (CSF) physiology for many decades, with deep expertise in the dynamics of brain fluids and the diverse functions of the choroid plexus/blood-CSF barrier (BCSFB). During his career, Malcolm greatly contributed to the advancement of our understanding of function of brain fluids and barriers. One could confidently say that Segal’s original work spurred resurgence in choroid plexus/CSF research in the twenty-first century. Of equal importance, his enthusiasm, kindness, and a unique, warm personality attracted to this field a large number of young researchers whom he took great pleasure in encouraging and supporting.

Malcolm Segal was born on the 1st of August 1937 in Goodmayes, Essex, UK, as the older son of Cyril and Eileen Segal. His father was a local pharmacist and expected his son to take over the family business, so Malcolm dutifully attended the School of Pharmacy in Bristol (1960) from where he graduated in 1963, becoming a registered pharmacist and a member of the Royal Pharmaceutical Society. However, to the disappointment of his father, Malcolm was not interested in pursuing a pharmacist’s career. Instead, he wanted to explore the functions of the human body and so enrolled in an undergraduate physiology course at University College London (UCL) in 1963. He ultimately was awarded a B.Sc. with honors in Physiology in 1966 and then joined Hugh Davson’s laboratory at UCL as a Ph.D. student. He was awarded a Ph.D. in Physiology in 1970 after presenting a thesis entitled *“A study of the factors affecting the exchange of electrolytes and other substances between the plasma and the central nervous systems.”* The thesis’s major experimental technique was ventriculo-cisternal perfusion (VCP) in rabbits, a method that Malcolm continued to use for decades. He employed VCP to study the entry of sodium from the blood into the CSF, as well as to investigate the factors controlling CSF secretion [1] and the role of CSF in distribution of extracellular markers [2]. Davson and Segal were also the first to develop the technique for measuring the CSF outflow resistance [3], a method that became commonly used by other laboratories worldwide.

Malcolm left Davson’s laboratory for Sherrington School of Physiology at St. Thomas’ Hospital Medical School, where he became a Lecturer and started his own research program. His initial focus was upon epithelial transport, including that at the choroid plexus/BCSFB, and the secretion and drainage of CSF. While the methods that Malcolm had initially used in Davson’s laboratory were valuable, they did not allow the investigator to discriminate between the transport processes at the choroid plexus/BCSFB from those at the blood–brain barrier (BBB). To address this issue, Malcolm adapted Mike Pollay’s method of the isolated sheep choroid plexus perfused in situ, modifying the preparation to allow bilateral perfusion, which allowed him to use both lateral ventricle choroid plexuses either together or separately. This method would ultimately be adopted by many other laboratories around the world and is still used today to measure transport of various molecules across the BCSFB.

One of the first projects that Malcolm embarked on in his new laboratory was to investigate why the sugar concentration in CSF is 50% lower than that in plasma. The data obtained by other groups [4–6], which used a variety of techniques, suggested that the choroid plexus generally pumps sugars out of the CSF. However, by employing isolated sheep choroid plexus perfused in situ, Malcolm and his Ph.D. student Rashid Deane were able to demonstrate that while there was a sodium-dependent efflux from the CSF to the blood, there was in fact a facilitated net entry of sugars in the opposite direction from the blood to the CSF. They also showed that the low levels of sugars in CSF resulted from the kinetic characteristics of the carrier-mediated entry processes across the choroid plexus epithelium into the CSF, and were not associated with efflux processes [7].

In the late seventies, important papers appeared from Segal’s lab—one on the effect of choroid plexus blood flow on the rate of CSF secretion [8], and another on CSF drainage mechanisms and the role of deep cervical lymph nodes in this process [9]. The latter paper was a seminal work, paving the way to a new area of research on brain immune response that was to be later pioneered by Helen Cserr and others [10, 11].

The next significant project that Segal’s laboratory took on was the investigation of the movement of amino acids across the choroid plexus/BCSFB. This project was particularly challenging because of two main issues—the accumulation of amino acids in erythrocytes and the existence of multiple carriers for amino acids. The former issue required a modification of the perfusate so that an erythrocyte-free buffered perfusate similar in composition to plasma could be used. The latter problem was addressed by replacing the original steady-state perfusion system with an indicator-dilution single-pass approach. This work was conducted by Jane Preston, another Ph.D. student in Segal’s lab. Together, they identified at least four different classes of amino acid transporters at the basolateral (blood-facing) side of the choroid plexus epithelium. In addition, they demonstrated that the net movement of amino acids was from the blood to the CSF. Interestingly, when the levels of amino acids were increased in the CSF, the flux reversed, changing the net direction from the CSF to the blood. This suggested a neuroprotective role for the choroid plexus in maintaining brain homeostasis [12–16].

With the arrival of new Ph.D. student Hameed Al-Sarraf in the mid to late nineties, the focus of Segal’s lab switched to developmental aspects of acidic amino acid transport across both the BCSFB and BBB. One of the hypotheses at the time was that the additional amino acids needed by the developing brain are provided simply by leaky brain barriers. Indeed, neonatal rats accumulate more amino acids in the CSF and brain compared to adult animals [17]. However, Segal’s group ultimately found that this phenomenon is not due to the leakiness of brain barriers in neonatal rats [18] but rather to a greater transporter capacity, elevated plasma amino acid levels, and a larger brain vascular space to deliver the amino acids in immature animals [19]. These factors, coupled with the lower rate of clearance of amino acids from the CSF and a slower rate of CSF secretion [20], were found to contribute to dynamically setting higher amino acid levels in the developing brain, in the presence of functional brain barriers.

In 1988, Malcolm Segal spent 3 months at Melbourne University, Australia in Gerhard Schreiber’s lab studying thyroxine (T4) carrier protein transthyretin (TTR), which is synthesized by the choroid plexus and secreted into the CSF. Upon returning to London, Malcolm continued this project in his lab with Jane Preston looking at the choroid plexus transport of thyroid hormones into the brain. The TTR project continued to be an important research focus in Segal’s lab until Malcolm’s retirement. Among the people working on this subject were Malcolm’s old friends and collaborators Rashid Deane, Wei Zheng, and Zoran Redzic, as well as his last Ph.D. student Nouhad Kassem. The resulting publications described the saturable uptake of triiodothyronine at the blood- and CSF-facing sides of the choroid plexus epithelium via a cyclic amino acid-inhabitable process [21], the effect of lead exposure on T4 entry into the CSF [22], as well as the confirmation of T4 distribution from the CSF into various brain regions using the original VCP technique [23]. It is somehow fitting that Malcolm’s final study with his colleagues published in 2009 [24] used the isolated perfused choroid plexus technique to explore the effect of aging on choroid plexus function, CSF secretion, and BCSFB integrity. This work contributed to the growing body of evidence that disturbances of fluid homeostasis and dysfunction of brain barriers are key features of age-related CNS disorders.

Malcolm made many important friendships along his scientific journey. During his stay in Melbourne in the late eighties, Malcolm also visited Howard Florey Institute of Experimental Biology and Medicine where he met Joanna and Adam Chodobski. These two scientists from Poland were investigating how dehydration affects CSF production and outflow resistance in sheep, but with the added dimension that the sheep they studied were conscious. Malcolm was amused by the experimental setup—sheep were carefully suspended in a sling, with one researcher keeping the sheep’s head still while the other researcher collected CSF samples. This brief encounter in Florey led not only to future collaboration with the Chodobskis but also provided the start to a long-lasting friendship. Upon returning from Australia to Poland in 1989, the Chodobskis stopped in London to visit Segal’s laboratory. Together, they came up with a research proposal that would allow them to conduct collaborative experiments on both sides of the English Channel. Indeed, after obtaining a grant from the Wellcome Trust, they would cross the Channel quite frequently for the next 2 years to work together. This collaboration resulted in three papers describing the roles of angiotensin II and arginine vasopressin in regulating CSF production and blood flow to the choroid plexus [25–27]. This time together also saw a deep friendship develop between the two families, so after the Chodobskis left Poland for the United States in 1991, Malcolm and his wife Mary continued to keep in close contact, with the two families visiting each other almost every year.

When in 1997/1998 the Chodobskis took on a new project of starting a Gordon Research Conference (GRC) on the *‘Barriers of the CNS*,*’* Malcolm was very supportive and became deeply involved in all aspects of its planning. The first *‘Barriers of the CNS’* GRC meeting was ultimately held at the Tilton School in New Hampshire in 1999, with Malcolm playing a key role. The 2020 *‘Barriers of the CNS’* now being organized will represent the 11th iteration of this highly impactful scientific meeting, widely regarded to be among the best in the field. The continued success of this meeting and the wonderful, collegial spirit of scientific exchange that is its hallmark are surely among Malcolm’s enduring legacies.

In 1994, Malcolm visited the Faculty of Medicine at the University of Belgrade, Serbia, where he met Zoran Redzic. Redzic was interested in homeostasis of nucleosides and nucleobases in the brain, especially the role of blood–brain interfaces in this process. Malcolm and Zoran found a mutual interest in science and this was the beginning of a long-lasting collaboration and friendship, with after-work intellectual and scientific discussions over a pint of *“Old Speckled Hen”* in *“The Archduke,”* family gatherings at his house in Pinner, and endless anecdotes about sheep and Malcolm’s involvement in his lab activities. During the period of 1995–2000, Malcolm and Zoran paid frequent visits to each other’s lab, working together on isolated perfused choroid plexus of the sheep [28, 29]. In 2001, Zoran was awarded a Wellcome Trust fellowship and moved to Malcolm’s lab at St. Thomas’ Hospital, where he and Malcolm then developed primary cultures of sheep choroid plexus epithelial cells to study transport and metabolism of nucleosides and nucleobases [30, 31]. They also used an in vivo indicator-dilution technique to study the influx and efflux of these molecules across the BBB in rats [32]. These studies shed new light on the role of the BCSFB and BBB in maintaining the homeostasis of nucleosides and nucleobases in the brain. They demonstrated a polarized distribution of nucleoside transporters at the BBB and in the choroid plexus epithelium, with concentrative transporters being located on the sides facing brain extracellular fluids (ISF and CSF, respectively) and the equilibrative transport being located on the opposite, blood-facing sides of these barriers. In addition, these studies showed that brain endothelial and choroid plexus epithelial cells constitute important enzymatic rather than physical barriers to the entry of nucleosides and nucleobases into the brain. Overall, these findings suggested that the brain barriers play a role in the removal rather than transport of nucleosides and nucleobases into the brain.

Malcolm’s special interest in the choroid plexus and BCSFB physiology also led to another interesting collaboration between his lab and the INSERM lab of Jean-François Ghersi-Egea and Nathalie Strazielle in Lyon, France, which specialized in choroid plexus cell cultures. Together with Sarah Thomas, who was working in Segal’s lab, Ghersi-Egea and Strazielle studied the delivery of nucleoside-derived antiviral drugs to the CSF using the combined in vivo and in vitro approaches. Their collaboration was instrumental in stimulating new interest in choroid plexus/CSF research, which at the time was in decline. Malcolm, Sarah, and the Lyon team organized the First International Workshop on Choroid Plexuses (CPWS) in Lyon in 2000 (Fig. 1). This meeting, which gathered scientists from five continents, focused on a variety of topics related to the choroid plexus/CSF biology, ranging from transport, CSF secretion, detoxification, endocrinology, and neuroimmunology in health and under pathological conditions, such as tumors, degenerative diseases, neuroinflammation, and hepatic encephalopathy.

**Fig. 1:**
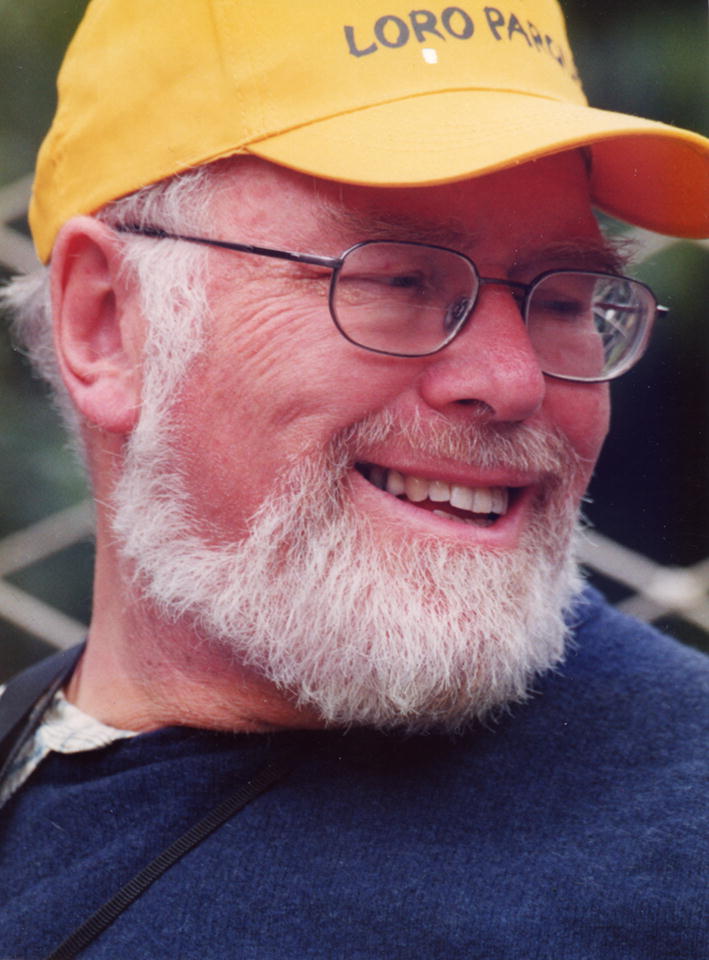
A photograph of Malcolm B. Segal taken by Zoran Redzic in 2000 during the First International Workshop on Choroid Plexuses in Lyon, France

During his professional career, Malcolm wrote numerous articles, and edited and co-authored many books, including the comprehensive cornerstone book on CSF titled *“The Physiology and Pathology of the Cerebrospinal Fluid”* [33]. The second edition of this book, which was published in 1996 [34], remains a highly valued source of authoritative information on CSF physiology and the brain barriers. Looking back at Malcolm’s contribution to this field, it is hard to escape the conclusion that his work was instrumental in a renaissance surrounding many diverse aspects of choroid plexus/CSF research.

In addition to his legacy as a researcher, an important aspect of Malcolm’s life to remember is that he greatly enjoyed teaching and working with students and young scientists. He mentored and trained eleven Ph.D. students from the UK and from abroad: Patricia Forman (1973), Arthur Gibson (1976), Ben Naidoo (1978), Paul Grinwald (1978), Rashid Deane (1982), Jane Preston (1989), Sarah Williams (now Thomas) 1994, Hameed Al-Sarraf (1996), Richard Egleton (1998), Kevin Smart (1999) and Nouhad Kassem (2004). As already mentioned, Malcolm collaborated with many laboratories all over the world and assisted many postdoctoral fellows, particularly those from the former Eastern Bloc countries, in the launching of their scientific careers. Malcolm frequently offered not only his expertise, but also the hospitality of his house and sincere friendship, an investment and commitment to his trainees that will never be forgotten. He was an exceptional teacher who taught almost all areas of physiology; however, he will above all be remembered for his outstanding knowledge and lectures on renal and epithelial physiology, as well as all manner of topics related to capillary dynamics.

Malcolm’s generosity of spirit and his impact upon the field, particularly his tireless encouragement and mentoring of new generations of scientists, continued unabated in the latter years of his career. One such young scientist to have been inspired and influenced by Malcolm’s unique ability to give so generously of himself was Robert Thorne. Thorne was working on his Ph.D. thesis when he presented a poster and first met Malcolm at the inaugural *‘Barriers of the CNS’* GRC meeting in 1999. Thorne had read a number of papers authored by Malcolm and was delighted to be presented with a unique opportunity to interact with him personally. Malcolm, in his own gracious way, embraced the occasion and, over the course of the following days, came to know that Robert and his wife Aparna were to be attending a scientific conference in Brighton, UK later the next spring. Learning that, Malcolm promptly invited them both to extend their visit and stay with Mary and himself in their house in Pinner, which they ultimately did. It is no exaggeration to say that this visit deeply touched Thorne. The combination of Mary and Malcolm’s hospitality, warm friendship, and nightly dinners together (served expertly by Mary), along with Malcolm’s arrangements over the course of several days for Thorne to meet and discuss science with colleagues at King’s College made a lasting impression. Robert and Malcolm continued their dialogue and friendship over the ensuing years, but the memory of this time in Malcolm’s home provided more inspiration to Robert than Malcolm could have ever known. Looking back, it is easy to see how Malcolm’s memory and spirit inspired Thorne and many other junior scientists to ultimately take on future roles of responsibility within the field, to build the global research community, and to work hard in supporting future generations of scientists, as Malcolm did with them.

Malcolm enjoyed sailing, good food, good wine, and good company, and combined his interests with love for teaching by running evening classes in wine tasting and sailing navigation, sometimes at the same time. Memories of this time spent together, his large personality, his warm, generous friendship, and the numerous anecdotes that made so many of us laugh to tears will always stay with us.

Malcolm Segal will be greatly missed. We will miss him for his contribution to science but also for his work as an educator and mentor to so many generations of young scientists. But most importantly, we will miss him as a kind and generous friend.

## Data Availability

Not applicable.
